# No protocol and no liability: a call for COVID crisis guidelines that protect vulnerable populations

**DOI:** 10.2217/cer-2020-0090

**Published:** 2020-07-24

**Authors:** Chiara Caraccio, Robert S White, Rohan Jotwani

**Affiliations:** ^1^Department of Microbiology & Immunology, Stanford University School of Medicine, Stanford, CA 94305, USA; ^2^Department of Anesthesiology, New York-Presbyterian/Weill Cornell Medicine, New York, NY 10065, USA

**Keywords:** COVID-19, health disparities, health policy, public health, resource allocation

## Abstract

The COVID-19 pandemic is revealing the unacceptable health disparities across New York City and in this country. The mortality rates of vulnerable and minority populations alone suggest a need to re-evaluate clinical decision making protocols, especially given the recently passed Emergency or Disaster Treatment Protection Act, which grants healthcare institutions full immunity from liability stemming from resource allocation/triage decisions. Here we examine the disparity literature against resource allocation guidelines, contending that these guidelines may propagate allocation of resources along ableist, ageist and racial biases. Finally, we make the claim that the state must successfully develop ones that ensure the just treatment of our most vulnerable.

The Coronavirus Disease 2019 (COVID-19) pandemic has disproportionately affected the most vulnerable populations in New York City and throughout the country. A total of 89% of those requiring hospitalization have underlying chronic health conditions [1]. New Yorkers over 75 constitute 60% of COVID-19 hospitalizations in the city, despite comprising only 6.4% of the population [2,3]. The CDC has reported that nationwide, 33% of hospitalized patients have been African American, despite the fact that only comprising 18% of the general population; in New York state, African Americans constitute 18% of deaths due to COVID-19, twice the number of their percentage of the population (9%) [4,5]. Low-income communities also suffer disproportionately: New York City ZIP Codes in the bottom 25% of average incomes constitute 36% of the city’s cases, whereas ZIP codes in the top 25% constitute under 10% of cases [6]. Yet while much of the evidence and commentary around disparity outcomes of COVID-19 addresses the differences in the underlying health status of those most severely affected, there is minimal discussion examining whether resource/triage allocation or liability policies may also be playing a role in exacerbating disparity outcomes [1–6].

COVID-19 has also shone a bright light on some gaping holes in emergency preparedness systems. Particularly, it has brought to light difficulties with insufficient medical supplies and rationing of resources, precipitating the previously unthinkable: how to triage resources in the case of an absolute deficit. The triage policies proposed by states and hospitals around the country have been nonuniform at best [7]. One common element among these heterogeneous policies is the tendency to further disadvantage the vulnerable populations already affected by COVID-19 (see Table 1 for a list of state policies and their distinguishing features).

**Table 1.** States’ resource allocation guidelines.

State	Does it have official resource allocation guidelines?^†^	COVID-specific?	Does it use SOFA -based scoring?	Notable features?	Ref.
Alabama	Yes, ‘Alabama Crisis Standards of Care’	No	No	Current version only includes clinical considerations. The Office for Civil Rights enacted a compliance review of Alabama’s 2010 guidelines which were originally in place during the COVID pandemic, which allegedly discriminated based on intellectual disability and strict age cut-offs.	[41]
Alaska	Yes	No	Yes	If necessary, people without severe underlying diseases with poor short-term prognoses would receive care before others.	[42]
Arizona	Yes, ‘Arizona Crisis Standards of Care Plan’	No	Yes	Arizona’s policy has not clashed with disability rights advocates, according to the Center for Public Integrity.	[43,44]
Arkansas	No				[44]
California	Yes, ‘California Severe acute respiratory syndrome coronavirus 2 (SARS-CoV-2) Pandemic Crisis Care Guidelines’	Yes	Yes	If necessary, younger people, people without chronic health conditions that limit life expectancy and vital healthcare workers would receive care before others.	[45]
Colorado	Yes, ‘CDPHE All Hazards Internal Emergency Response and Recovery Plan ANNEX B: Colorado Crisis Standards of Care Plan 5 April 2020’	Yes	Yes	If necessary, younger people, people without chronic health conditions that limit life expectancy, vital healthcare workers and first responders, pregnant women and primary caregivers would receive care before others.	[46]
Connecticut	Yes, ‘Standards of Care: Providing HealthCare During a Prolonged Public Health Emergency’	No	No	Recommends the American College of Chest Physicians triage system over SOFA.	[47]
Delaware	No				
Florida	Yes, ′Pandemic Influenza: Triage and Scarce Resource Allocation Guidelines’	No	Yes	If necessary, people without severe underlying diseases with poor short-term prognoses would receive care before others.	[48]
Georgia	No				
Hawaii	No				
Idaho	No				[44]
Illinois	No				
Indiana	Yes – ‘Crisis Standards of Patient Care Guidance with an Emphasis on Pandemic Influenza: Triage and Ventilator Allocation Guidelines’	No	Yes	Terminal disease with a life expectancy of <6 months is on list of exclusion criteria. Indiana’s COVID-19 Joint Information Center has stated that this guidance document is not part of the COVID response plan. Current plans do not contain alternate triage resource allocation guidelines.	[44,49]
Iowa	Yes, ‘An Ethical Framework for Use in a Pandemic’	No	Yes	If necessary, suggests that younger and nonimmunocompromised persons would receive care before others, based on ‘survivability’. Suggests healthcare workers might be prioritized.	[50]
Kansas	Yes, ‘Guidelines for the Use of Modified HealthCare Protocols in Acute Care Hospitals During Public Health Emergencies’	No	Yes	Severe, advanced chronic disease with a life expectancy of <6 months, advanced untreatable neuromuscular disease and metastatic malignant disease with poor prognosis are on list of exclusion criteria.	[51]
Kentucky	Yes, ‘Crisis Standards of Care: Guidance for the Ethical Allocation of Scarce Resources during a Community-Wide Public Health Emergency’	No	Yes	High 1-year mortality probability and requirement of a ‘larger-than-normal’ amount of resources are on list of exclusion criteria	[52]
Louisiana	Yes, ‘State Hospital Crisis Standard of Care Guidelines in Disasters’	No	Yes	Known severe dementia, advanced untreatable neuromuscular disease ‘requiring assistance with activities of daily living or requiring chronic ventilator support’ are on list of exclusion criteria.	[53]
Maine	No				
Maryland	Yes, ‘Maryland Framework for the Allocation of Scarce Life-sustaining Medical Resources in a Catastrophic Public Health Emergency’	No	Yes	If necessary, children, young persons (based on age brackets), patients with higher prospects for long-term survival and pregnant women in their third trimester with healthy fetuses would receive care before others.	[54]
Massachusetts	Yes, ‘Crisis Standards of Care Planning Guidance for the COVID-19 Pandemic’	Yes	Yes	If necessary, people without chronic health conditions that limit life expectancy, vital healthcare workers, pregnant women and young persons (based on age brackets), would receive care before others.	[55]
Michigan	Yes, ‘Guidelines for Ethical Allocation of Scarce Medical Resources and Services During Public Health Emergencies in Michigan’	No		If necessary, essential workers (including healthcare workers, first responders, public health scientists, personnel key to public safety, for example, police, fire, military etc. and personnel key to critical infrastructure, for example, energy grid, telecommunications etc.) would receive care before others. Suggests that considerations of age and disability-adjusted life years might be used as additional criteria by decision makers.	[56]
Minnesota	Yes, ‘For the Good of Us All: Ethically Rationing Health Resources in Minnesota in a Severe Influenza Pandemic’	No	Yes	If necessary, younger people and key workers – based both on utilitarian considerations and ‘reciprocity obligations’ – would receive care before others	[57]
Mississippi	No – ‘Mississippi Pandemic Influenza Incident Annex’ does not contain allocation criteria	No	n/a		[58]
Missouri	No				
Montana	No				
Nebraska	No				
Nevada	Yes, ‘Nevada Crisis Standards of Care Plan’ (COVID-specific additions in ‘Crisis Standards of Care Crisis Level Guidance for COVID-19’)	Yes	Yes	Nevada’s policy does not have any of the problems that disability rights advocates have decried in other states, according to the Center for Public Integrity.	[44,59]
New Hampshire	No				
New Jersey	No				[44]
New Mexico	Yes, ‘New Mexico Crisis Standards of Care Plan’	No	Yes	New Mexico’s policy does not have any of the problems that disability rights advocates have decried in other states, according to the Center for Public Integrity.	[60]
New York	Yes, ‘Ventilator Allocation Guidelines’	No	Yes	If necessary, minors would receive care before others.	[10]
North Carolina	Yes, ‘Stockpiling Solutions: North Carolina’s Ethical Guidelines for an Influenza Pandemic’	No	n/a	North Carolina’s policy does not have any of the problems that disability rights advocates have decried in other states, according to the Center for Public Integrity.	[61]
North Dakota	No				[44]
Ohio	No				
Oklahoma	Yes, ‘Hospital Crisis Standards of Care’	No	Yes	If necessary, people without chronic health conditions that limit life expectancy or that necessitate ongoing resource demand (e.g., home oxygen dependent, dialysis dependent) would receive care before others.	[62]
Oregon	Yes, ‘Oregon Crisis Care Guidance’	No	Yes	If necessary, people without chronic health conditions that limit life expectancy to <6–12 months would receive care before others. Viability of the fetus in the case of pregnant women and amount of resources needed per individual might also be considered.	[63]
Pennsylvania	Yes, ‘Interim Pennsylvania Crisis Standards of Care for Pandemic Guidelines’	Yes	Yes	If necessary, people without chronic health conditions that limit life expectancy (including dementia, malignancies with a <10 year expected survival, etc.), pregnant women with viable fetuses, key healthcare personnel and young persons (based on age brackets), would receive care before others.	[64]
Rhode Island	No				
South Carolina	Yes, ‘South Carolina Prepares for Pandemic Influenza: An Ethical Perspective’	No	Yes	If necessary, people without chronic health conditions that limit life expectancy and young persons (based on age brackets) would receive care before others.	[65]
South Dakota	No				
Tennessee	Yes, ‘Guidance for the Ethical Allocation of Scarce Resources during a Community-Wide Public Health Emergency as Declared by the Governor of Tennessee’	No	Yes	Advanced untreatable neuromuscular disease ‘requiring assistance with activities of daily living or requiring chronic ventilator support’ is on list of exclusion criteria. Disability Advocates have filed a formal federal complaint about Tennessee's policy.	[44,66]
Texas	Yes, ‘North Texas Mass Critical Care Guidelines Document Hospital and ICU Triage Guidelines for adults’ (not officially state adopted)	No	Yes	Advanced untreatable neuromuscular disease ‘requiring assistance with activities of daily living or requiring chronic ventilator support’ and severe dementia are on list of exclusion criteria.	[67]
Utah	Yes, ‘Utah Pandemic Influenza Hospital and ICU Triage Guidelines’	No	Yes	Advanced untreatable neuromuscular disease ‘requiring assistance with activities of daily living or requiring chronic ventilator support’ and severe dementia are on list of exclusion criteria.	[68]
Vermont	Yes, ‘Vermont Crisis Standards of Care Plan’	No	Yes	If necessary, people without chronic health conditions that limit life expectancy (e.g., cystic fibrosis) and require ongoing resource demand would receive care before others.	[69]
Virginia	No				[44]
Washington	Yes, ‘Scarce Resource Management & Crisis Standards of Care’	No	Yes	Disability advocates have filed a formal complaint about Washington’s policy.	[44,70]
West Virginia	No				
Wisconsin	Yes, ‘Wisconsin Adult Ventilator Guidelines’ (not officially state adopted)	No	Yes	If necessary, people without chronic health conditions that limit life expectancy (e.g., cystic fibrosis) and require ongoing resource demand (e.g., severe stroke, severe dementia, etc.) would receive care before others.	[71]
Wyoming	No				[44]

† No: No policy available or undisclosed.

SOFA: Sequential organ failure assessment.

The most widely-commented on form of discrimination has been that of policies that disadvantage the disabled community. A recent study conducted by the Association of Bioethics Program Directors (ABPD) surveying the ventilator triage protocols of hospitals around the country found that 38.5% of hospital protocols factor resource conservation into their protocol criteria, designating that individuals in need of increased clinical attention and resource-use are a lower priority [7]. Only 26.9% of policies specified that allocation decisions should not be based on disability and some of these policies themselves included decision criteria that would disproportionately disqualify the disabled community [7]. Disability Rights New York, an advocacy group for persons with disabilities in New York State, has previously filed a complaint against the New York Department of Health for its 2015 ventilator triage policy, which failed to specify that allocation decisions ought exclude disability. The complaint argues that without explicit instruction urging awareness against implicit bias, hospitals will disproportionately categorize disabled persons as having conditions that disqualify them from ventilator access, even when these conditions do not impact their short-term potential to survive [8]. Advocates have also argued that submitting chronically disabled persons to the same clinical litmus tests for ventilator allocation as healthy persons, such as difficulties at the time of extubation, denies equal access of healthcare facilities to the disabled community [8,9].

Less attention has been paid to age-based discrimination. New York’s 2015 guidelines acknowledge the inequity of factoring age into allocation decisions, but establish a ‘tie-breaker’ in which children under the age of 18 will be given priority over adults in the case that both would benefit equally from ventilator use [10]. A total of 50% of policies assessed in the ABPD study utilized age in their criteria [7].

Discrimination against racial minorities may be a feature of any policies that include the presence of comorbidities in their decision criteria. African American patients are three-times more likely to have kidney failure than their white counterparts, nearly twice as likely to suffer congestive heart failure, 40% more likely to have high blood pressure and less likely to have that blood pressure under control, have higher reported rates of sepsis and are 50% more likely to have chronic liver disease [11–17]. Hispanic patients are 1.5-times more likely to have kidney failure than their white counterparts, 1.5-times more likely to suffer congestive heart failure, have higher reported rates of sepsis and are twice as likely to have chronic liver disease [11,13–19]. Per the ABPD study, 95% of ventilator triage policies utilize the sequential organ failure assessment scores to determine allocation of resources, where higher scores often tend to correlate with worse outcomes and increased baseline comorbidities [7,10]. In light of this, there has been public outcry by physicians that these policies inevitably bias resource allocation away from minority populations with higher likelihood of worse initial assessments that underscores these comorbidities [20]. Low-income populations, who also suffer a higher rate of comorbidities, may also be negatively impacted by these policies compared with their wealthier counterparts [21].

Substantive protocol aside, certain procedural features also need to be examined to ensure just treatment. Only 7.7% of hospitals require allocation decisions to be blinded [7]. On the one hand, granting decision-makers knowledge of the patient’s nonclinical characteristics may introduce the possibility of implicit bias playing a role in triage decisions, especially given that biases in medicine have been shown to be exacerbated in high-stress environments [22–25]. Alternatively, it may be that identity-blind triage criteria do more harm than good; by ignoring the reality that social determinants of health disproportionately disadvantage minority communities, triage criteria that seek to maximize lives saved without correcting for these disadvantages will further deprioritize the lives of the most at-risk groups [26]. Balancing consideration of comorbidities that matter to overall survival with a just and equitable allocation of resources continues to prove difficult for many institutions [20]. Further, the historic difficulty behind successfully incorporating factors such as race into healthcare policy to increase equitable outcomes highlights the acute need for meticulously thought-out policies, developed according to input from physicians and experts well versed in equity issues and from diverse backgrounds [26].

The protocols mentioned above have been defended on the grounds of providing the greatest public benefit during the pandemic. The New York 2015 guidelines, specifically state the aim of these protocols are to “*[encourage] allocation practices best suited to maximizing public health*” [10]. Undoubtedly, preserving public health during a pandemic is crucial, but protocols that exacerbate disparities based on race, age or disability do not serve the public interest *a priori*. Yet, the appeal to public benefit to justify unjust treatment of the marginalized is not a new concept. Historically, charitable hospitals have tried to claim total immunity from civil or criminal liability stemming from malpractice suits by arguing that their charitable trusts were designed to be used to continue treating patients for free, rather than to compensate poor patients who had suffered from negligent treatment (*Silva v. Providence Hospital of Oakland, England v. Hospital of Good Samaritan, Wilmington General Hospital v. Manlove*) [27–29]. In the landmark case *Tunkl v. Regents of the University of California* (1963), the court decided that UCLA Medical Center could not force indigent patients to sign a contract releasing UCLA from all malpractice liability in exchange for treatment, establishing that the most vulnerable in our population shall not have their rights denied [30,31]. The majority opinion explains that ‘public interest’ is not something that can be narrowly defined; in “*the integrated…society of today, structured upon mutual dependency….prearranged exculpation from [a hospital’s] negligence…necessarily affects the public interest*” [30]. The same rings truer today than ever: in an interdependent society, prearranged exculpation from harms to our most vulnerable is itself a threat to the public interest.

But nearly 60 years after *Tunkl*, similar ethical quandaries have been tied to the COVID pandemic. On 2 April 2020, as a part of the 2020–21 New York State budget, the ‘Emergency or Disaster Treatment Protection Act’ (EDTPA) was signed into law [32]. The Act grants healthcare workers, including physicians, administrators and hospital managers, immunity from criminal and civil liability for harms and damages resulting from the COVID-19 crisis. Immunity will not be granted for acts constituting willful or intentional criminal misconduct, gross negligence, reckless misconduct or intentional infliction of harm. However, EDTPA states explicitly that acts, omissions and decisions resulting from resource or staffing shortages will not be considered to fall into any of those aforementioned categories (§ 3082 2) [32].

In other words, the act constructs prearranged exculpation from a hospital’s negligence. The immunity granted from the threat of a malpractice suit to healthcare workers and volunteers treating COVID-19 patients with limited resources and at potential risk to their own safety is a widely praised development [33–35]. However, the breadth of roles granted immunity and the wording of the act raises concerns about the fate of marginalized communities in the case of the COVID-19 situation worsening. Past literature that has called for physician immunity during public health emergencies for all but gross negligence and intentional misconduct, as the EDTPA does, has still maintained that certain acts, such as extubation of one patient to benefit another, should not be entitled to immunity because they would fall under the gross negligence or the intentional misconduct umbrella [36]. The EDTPA’s explicit protection of triage/resource allocation decisions, given the concerning ethical implications of the existing protocols, has major implications for preventing and holding institutions accountable for disparity outcomes.

There is an explicit difference between *Tunkl* and related cases and the COVID pandemic and this difference is the key to this looming ethical problem: hospitals are not now seeking to disenfranchise their patients, but rather the opposite. During this crisis, healthcare and allied hospital essential workers have shown that they will risk their own lives to help their patients. Many of the unorganized and discriminatory policies that hospital triage protocols around the country have exhibited may be a symptom of difficult decision making during an all-encompassing pandemic, not of malintent. According to the ABPD study, 50% of hospitals nationwide have not had time to draft official policy at all; this percentage is likely much higher across all hospitals, since the ABPD study only surveyed hospitals with Bioethics programs, which may be more likely to have the appropriate infrastructure to create such policies in the first place [7]. According to the 2015 New York guidelines, hospitals have “*stressed that they are eager to follow State-level guidance*” and have “*expressed a preference for State guidance over drafting their own policies*” [10].

The solution, then, is clear: the state must come forward with a protocol that adequately secures the rights of the vulnerable and disseminate it to our hospitals. Vulnerable populations deserve just treatment and our healthcare workers deserve the immunity that the EDTPA grants them: these two just deserts are only in tension when our policies discriminate against the vulnerable and the state leaves them with no recourse to be compensated for the damages they suffer.

Unfortunately, the state has not done this. The 2015 New York protocols are plagued with issues; in addition to their discriminatory clauses, this protocol has not been updated to reflect decision-making more likely to occur with COVID-19, such as clinical judgement concerning likelihood of multiorgan failure or predicting length of mechanical ventilatory needs prior to intubation. However, even if this protocol were perfect, the greater issue is that it has been abandoned by state leadership. Andrew Cuomo declared ‘there’s no protocol’ when asked about triage policy for resource management and a department of health spokesperson, directly contrary to the existence of the 2015 guidelines, stated explicitly ‘we have no guidelines’ when asked [37,38].

The importance of having a standardized framework for triage decisions is not merely a matter of ensuring that each document contains just policies. Standardization is in itself a virtue during a crisis: the CDC states that, “*making decisions about ventilator distribution and triage using a standard framework for incident management creates a clear hierarchy of accountability and responsibility, facilitates consistent communication and helps minimize differential treatment of patients*” [11]. Across medicine the use of standardized protocols has been shown to decrease medical provider implicit bias and has been shown to decrease healthcare disparities [39,40]. Crucially, a standardized document would also ensure that each hospital has a robust triage decisions appeals process in place, since the total immunity granted to healthcare workers and institutions by the EDTPA renders legal avenues of appeal moot. According to the ABPD study, less than 70% of hospitals have appeals processes in place and only 61.5% specify methods for retrospectively reviewing their own decisions to ensure their policies are being implemented fairly [7].

In *Tunkl v. Regents of the University of California*, the state came down on the side of the vulnerable against the interests of the hospital. Thankfully, today there does not need to be any weighing of the rights of marginalized communities against the rights of healthcare workers during this crisis. Today we see our healthcare workers risking their lives for their communities; the liability protection that the EDTPA grants them is welcome and just. The state must now provide and actively promote a framework that ensures that our physicians can continue providing for every community, especially the marginalized. Creating a truly just policy will likely entail working under multidisciplinary collaboration with healthcare workers, bioethicists and other healthcare professionals with the goal of protecting vulnerable and at risk populations. Indeed, time is running out.

## References

[1] NPR. Who's hit hardest by COVID-19? why obesity, stress and race all matter (2020). www.npr.org/sections/healthshots/2020/04/18/835563340/whos-hit-hardest-by-covid-19-why-obesity-stress-and-race-allmatter

[2] Woodward A, Gould S, Kiersz A Cuomo says “the worst is over” in New York. But hospitalization rates for New York City's oldest coronavirus patients went up in the last week. Business insider (2020). www.businessinsider.com/new-york-city-coronavirus-cases-deaths-hospitalizations-by-age-chart-2020-3?r=US&IR=T

[3] NYCdata. Age and sex distribution - by county (2020). www.baruch.cuny.edu/nycdata/population-geography/age_distribution.htm

[4] ABC News. Coronavirus is disproportionately killing the black community. Here's what experts say can be done about it. ABC News (2020). https://abcnews.go.com/Politics/coronavirus-disproportionately-killing-black-communityexperts/story?id=70011986

[5] Eyewitness News ABC7. Minorities hit harder by COVID-19, data shows (2020). https://abc7ny.com/black-americanscoronavirus-nyc-update-corona-virus/6086065/

[6] Time. These graphs show how COVID-19 is ravaging New York City's low-income neighborhoods (2020). https://time.com/5821212/coronavirus-low-income-communities/

[7] Antommaria AHM, Gibb TS, McGuire AL Ventilator triage policies during the COVID-19 pandemic at U.S. hospitals associated with members of the Association of Bioethics Program Directors. Ann. Intern. Med. (2020) (Epub ahead of print). 10.7326/M20-1738 PMC7207244 32330224

[8] Ventilator rationioning - OCR complaint FINAL.pdf. (2020). www.dropbox.com/s/h3hjktdvz3qxes3/2020.04.07%20-%20Ventilator%20Rationioning%20-%20OCR%20Complaint%20FINAL.pdf?dl=0

[9] The New York Times. I will not apologize for my needs (2020). www.nytimes.com/2020/03/23/opinion/coronavirus-ventilators-triage-disability.html

[10] Zucker H, Adler K, Berens D, Bleich RJ, Brynner R, Butler KA New York State Task Force on life and the law. New York State Department of Health. Ventilator Allocation Guidelines (2015). www.health.ny.gov/regulations/task_force/reports_publications/docs/ventilator_guidelines.pdf

[11] National Kidney Foundation. Race, ethnicity & kidney disease (2016). www.kidney.org/atoz/content/minorities-KD

[12] Bahrami H, Kronmal R, Bluemke DA Differences in the incidence of congestive heart failure by ethnicity. Arch. Intern. Med. 168(19), 2138–2145 (2008). 18955644 10.1001/archinte.168.19.2138 PMC3038918

[13] Barnato AE, Alexander SL, Linde-Zwirble WT, Angus DC Racial variation in the incidence, care and outcomes of severe sepsis. Am. J. Respir. Crit. Care Med. 177(3), 279–284 (2008). 17975201 10.1164/rccm.200703-480OC PMC2720103

[14] Dombrovskiy VY, Martin AA, Sunderram J, Paz HL Occurrence and outcomes of sepsis: influence of race. Crit. Care Med. 35(3), 763–768 (2007). 17255870 10.1097/01.CCM.0000256726.80998.BF

[15] Martin GS, Mannino DM, Eaton S, Moss M The epidemiology of sepsis in the United States from 1979 through 2000. N. Engl. J. Med. 348(16), 1546–1554 (2003). 12700374 10.1056/NEJMoa022139

[16] Mayr FB, Yende S, Linde-Zwirble WT Infection rate and acute organ dysfunction risk as explanations for racial differences in severe sepsis. JAMA 303(24), 2495–2503 (2010). 20571016 10.1001/jama.2010.851 PMC3910506

[17] US Department of Health and Human Services Office of Minority Health. Chronic liver disease and African Americans (2020). www.minorityhealth.hhs.gov/omh/browse.aspx?lvl=4&lvlid=17

[18] US Department of Health and Human Services Office of Minority Health. Heart disease and African Americans (2020). www.minorityhealth.hhs.gov/omh/browse.aspx?lvl=4&lvlid=19

[19] US Department of Health and Human Services Office of Minority Health. Chronic liver disease and Hispanic Americans (2020). www.minorityhealth.hhs.gov/omh/browse.aspx?lvl=4&lvlid=62

[20] NPR. Opinion: US must avoid building racial bias into COVID-19 emergency guidance (2020). www.npr.org/sections/healthshots/2020/04/21/838763690/opinion-u-s-must-avoid-building-racial-bias-into-covid-19-emergency-guidance

[21] Akinyemiju T, Jha M, Moore JX, Pisu M Disparities in the prevalence of comorbidities among US adults by state Medicaid expansion status. Prev. Med. 88, 196–202 (2016). 27095325 10.1016/j.ypmed.2016.04.009 PMC4902718

[22] Sabin J, Nosek BA, Greenwald A, Rivara FP Physicians' implicit and explicit attitudes about race by MD race, ethnicity and gender. J. Health Care Poor Underserved 20(3), 896–913 (2009). 19648715 10.1353/hpu.0.0185 PMC3320738

[23] Maina IW, Belton TD, Ginzberg S, Singh A, Johnson TJ A decade of studying implicit racial/ethnic bias in healthcare providers using the implicit association test. Soc. Sci. Med. 199, 219–229 (2018). 28532892 10.1016/j.socscimed.2017.05.009

[24] Stepanikova I Racial-ethnic biases time pressure and medical decisions. J. Health Soc. Behav. 53(3), 329–343 (2012). 22811465 10.1177/0022146512445807

[25] Dyrbye L, Herrin J, West CP Association of racial bias with burnout among resident physicians. JAMA Netw. Open 2(7), e197457 (2019). 31348503 10.1001/jamanetworkopen.2019.7457 PMC6661712

[26] Cleveland Manchanda E, Couillard C, Sivashanker K Inequity in crisis standards of care. N. Engl. J. Med. (2020) (Epub ahead of print). 10.1056/NEJMp2011359 32402154

[27] Justia US Law. Silva v. Providence Hospital of Oakland (2020). https://law.justia.com/cases/california/supreme-court/2d/14/762.html

[28] Justia US Law. England v. Hospital of Good Samaritan (2020). https://law.justia.com/cases/california/supreme-court/2d/14/791.html

[29] Justia US law.Wilmington General Hospital v. Manlove (2020). https://law.justia.com/cases/delaware/supreme-court/1961/174-a-2d-135-3.html

[30] Justia US law. Tunkl v. Regents of University of California (2020). https://law.justia.com/cases/california/supreme-court/2d/60/92.html

[31] Rothstein MA Malpractice immunity for volunteer physicians in public health emergencies. J. Law Med. Ethics 38(1), 149–153 (2010). 20446993 10.1111/j.1748-720X.2010.00475.x PMC3032940

[32] Winget, Spadafora & Schwartzberg, LLP. New York State Legislature enacts Emergency Disaster Treatment Protection Act (2020). https://wssllp.com/new-york-state-legislature-enacts-emergency-disaster-treatment-protection-act/

[33] The New York Times. Opinion: doctors need room to make tough decisions on coronavirus care (2020). www.nytimes.com/2020/04/04/opinion/coronavirus-doctors-lawsuitsprosecution.html

[34] American Medical Association. AMA to governors: physicians need COVID-19 liability protections (2020). www.ama-assn.org/practice-management/sustainability/ama-governors-physicians-need-covid-19-liability-protections

[35] Pittsburgh Post-Gazette. Doctors to Gov. Wolf: don't thank us, protect us (2020). www.post-gazette.com/opinion/Op-Ed/2020/05/06/Lawrence-John-doctors-Tom-Wolf-protect-us/stories/202005060020

[36] Hoffman S Responders' responsibility: liability and immunity in public health emergencies. Georgetown Law J. 96(6), 1913–1970 (2007).

[37] The New York Times. Patient has virus and serious cancer. should doctors withhold ventilator? (2020). www.nytimes.com/2020/04/01/nyregion/coronavirus-doctors-patients.html

[38] The New Yorker. Who gets a ventilator? (2020). www.newyorker.com/magazine/2020/04/20/who-gets-a-ventilator

[39] Rozental O, White RS, Weinberg R Role of adherence to enhanced recovery after surgery programs in mitigating health care disparities. JAMA Surg. 155(1), 91–92 (2020). 10.1001/jamasurg.2019.3486 31553426

[40] Wahl TS, Goss LE, Morris MS Enhanced Recovery After Surgery (ERAS) eliminates racial disparities in postoperative length of stay after colorectal surgery. Ann. Surg. 268(6), 1026–1035 (2018). 28594746 10.1097/SLA.0000000000002307 PMC9945468

[41] HHS.gov. OCR reaches early case resolution with alabama after it removes discriminatory ventilator triaging guidelines (2020). www.hhs.gov/about/news/2020/04/08/ocr-reaches-early-case-resolution-alabama-after-it-removes-discriminatory-ventilator-triaging.html

[42] Minnesota Department of Health. Patient care strategies for scarce resource situations (2020). www.health.state.mn.us/communities/ep/surge/crisis/standards.pdf

[43] Arizona Department of Health Services. Arizona crisis standards of care plan (2018). www.azdhs.gov/documents/preparedness/emergency-preparedness/response-plans/azcsc-plan.pdf

[44] Center for Public Integrity. State policies may send people with disabilities to the back of the line for ventilators (2020). https://publicintegrity.org/health/coronavirus-and-inequality/state-policies-may-send-people-with-disabilities-to-the-back-of-the-line-for-ventilators/

[45] California Department of Public Health. California SARS-CoV-2 pandemic crisis care guidelines. https://www.cdph.ca.gov/Programs/CID/DCDC/CDPH%20Document%20Library/COVID-19/California%20SARS-CoV-2%20Crisis%20Care%20Guidelines%20-June%208%202020.pdf

[46] Colorado Department of Public Health and Environment. CDPHE all hazards internal emergency response and recovery plan. ANNEX B: Colorado crisis standards of care plan (2018). https://cha.com/wp-content/uploads/2018/10/Crisis-Standards-of-Care-05102018-FINAL.pdf

[47] Standards of Care Workgroup CT Department of Public Health. Standards of care: providing health care during a prolonged public health emergency (2010). https://portal.ct.gov/-/media/Departments-and-Agencies/DPH/dph/legal/StandardsofCarefinalpdf.pdf?la=en

[48] Florida Department of Health. Pandemic influenza technical advisory committee. pandemic influenza: triage and scarce resource allocation guidelines (2011). https://bioethics.miami.edu/_assets/pdf/about-us/special-projects/acs-guide.pdf

[49] Indiana State Department of Health. Crisis standards of patient care guidance with an emphasis on pandemic influenza: triage and ventilator allocation guideline (2014). https://emeraldcoasthcc.org/sites/emeraldcoasthcc.site/files/indiana-crisis-standards-of-care-2014.pdf

[50] Iowa Department of Public Health. Report of the Iowa pandemic influenza ethics committee. an ethical framework for use in a pandemic (2007). http://publications.iowa.gov/17889/1/panflu_ehtical_guidelines_manual.pdf

[51] Kansas Department of Health and Environment. Guidelines for the use of modified health care protocols in acute care hospitals during public health emergencies (2009). www.kdheks.gov/cphp/download/Crisis_Protocols.pdf

[52] Kentucky Department for Public Health. Crisis standards of care: guidance for the ethical allocation of scarce resources during a community-wide public health emergency (2020). www.documentcloud.org/documents/6824977-Kentucky-Crisis-Standards-of-Care

[53] Louisiana Department of Health & Hospitals. ESF-8 health & medical section state hospital crisis standard of care guidelines in disasters (2011). https://cdn.ymaws.com/www.lhaonline.org/resource/resmgr/imported/Louisiana%20CSOC%20Guidelines%20in%20Disasters.pdf

[54] Maryland framework for the allocation of scarce life-sustaining medical resources in a catastrophic public health emergency (2017). www.bioethics.net/wp-content/uploads/2020/03/Daugherty-Maryland-framework-PH-emergency-2017.pdf?x41592

[55] Massachusetts Executive Office of Health and Human Services, Department of Public Health. Crisis standards of care planning guidance for the COVID-19 pandemic (2020). https://d279m997dpfwgl.cloudfront.net/wp/2020/04/CSC_April-7_2020.pdf

[56] State of Michigan, Department of Community Health, Office of Public Health Preparedness. Guidelines for ethical allocation of scarce medical resources and services during public health emergencies in Michigan (2012). www.mimedicalethics.org/Documentation/Michigan%20DCH%20Ethical%20Scarce%20Resources%20Guidelines%20v2%20rev%20Nov%202012.0.pdf

[57] Vawter DE, Garrett JE, Gervais KG For the good of us all: ethically rationing health resources in Minnesota in a severe influenza pandemic (2010). www.health.state.mn.us/communities/ep/surge/crisis/ethics.pdf

[58] Mississippi Department of Public Health. Mississippi pandemic influenza incident annex (2019). https://msdh.ms.gov/msdhsite/_static/resources/2944.pdf

[59] Nevada Division of Public and Behavioral Health. Nevada crisis standards of care (CSC) plan (2017). https://files.asprtracie.hhs.gov/documents/nv-csc-plan-070317–final-508.pdf

[60] New Mexico Department of Health. New Mexico crisis standards of care plan (2018). www.nmhealth.org/publication/view/plan/4877/

[61] North Carolina Institute of Medicine and North Carolina Department of Health and Human Services, Division of Public Health. Stockpiling solutions: North Carolina's ethical guidelines for an influenza pandemic (2007). http://nciom.org/stockpiling-solutions-north-carolinas-ethical-guidelines-for-an-influenza-pandemic/

[62] Oklahoma State Department of Public Health. Emergency preparedness and response service. hospital crisis standards of care. www.ok.gov/health2/documents/Hospital%20Crisis%20Standards%20of%20Care.pdf

[63] Oregon Department of Health. Oregon crisis care guidance (2018). www.theoma.org/CrisisCare

[64] Pennsylvania Department of Health and The Hospital Health System Association of Pennsylvania. Interim Pennsylvania crisis standards of care for pandemic guidelines (2020). https://int.nyt.com/data/documenthelper/6850-pennsylvania-triage-guidelines/02cb4c58460e57ea9f05/optimized/full.pdf#page=1

[65] South Carolina Influenza Ethics Taskforce, South Carolina Department of Health and Environmental Control. South Carolina prepares for pandemic influenza: an ethical perspective (2009). www.scdhec.gov/sites/default/files/Library/CR-009538.pdf

[66] Tennessee Altered Standards of Care Workgroup, Tennessee Department of Health and Tennessee Hospital Association. Guidance for the ethical allocation of scarce resources during a community-wide public health emergency as declared by the governor of tennessee (2016). www.shelbytnhealth.com/DocumentCenter/View/847/2016-Guidance-for-the-Ethical-Allocation-of-Scarce-Resources

[67] North Texas Mass Critical Care Task Force. North Texas mass critical care guidelines document hospital and ICU triage guidelines for ADULTS (2014). www.dallas-cms.org/tmaimis/dcms/assets/files/communityhealth/MCC/GuidelinesAdult_JAN2014.pdf

[68] Utah Hospitals and Health Systems Association for the Utah Department of Health. Utah pandemic influenza hospital and ICU triage guidelines (2009). http://pandemicflu.utah.gov/plan/med_triage081109.pdf

[69] Vermont Department of Health. Vermont crisis standards of care plan (2019). www.healthvermont.gov/sites/default/files/documents/pdf/VT%20CSC%20Plan%2007-23-2019%20Final.pdf

[70] Washington State Department of Health. Scarce resource management & crisis standards of care (2020). https://nwhrn.org/wp-content/uploads/2020/03/Scarce_Resource_Management_and_Crisis_Standards_of_Care_Overview_and_Materials-2020-3-16.pdf

[71] Wisconsin Hospital Association. Wisconsin adult ventilator guidelines (2008). www.documentcloud.org/documents/6824990-Wisconsin-Adult-ventilator-guidelines-From

